# Supplementary figures and images for: Automated registration and clustering for enhanced localization atomic force microscopy of flexible membrane proteins
Source: PLoS Comput Biol. 2025 Dec 1;21(12):e1013277. doi: 10.1371/journal.pcbi.1013277 (PMC12680352; doi:10.1371/journal.pcbi.1013277)

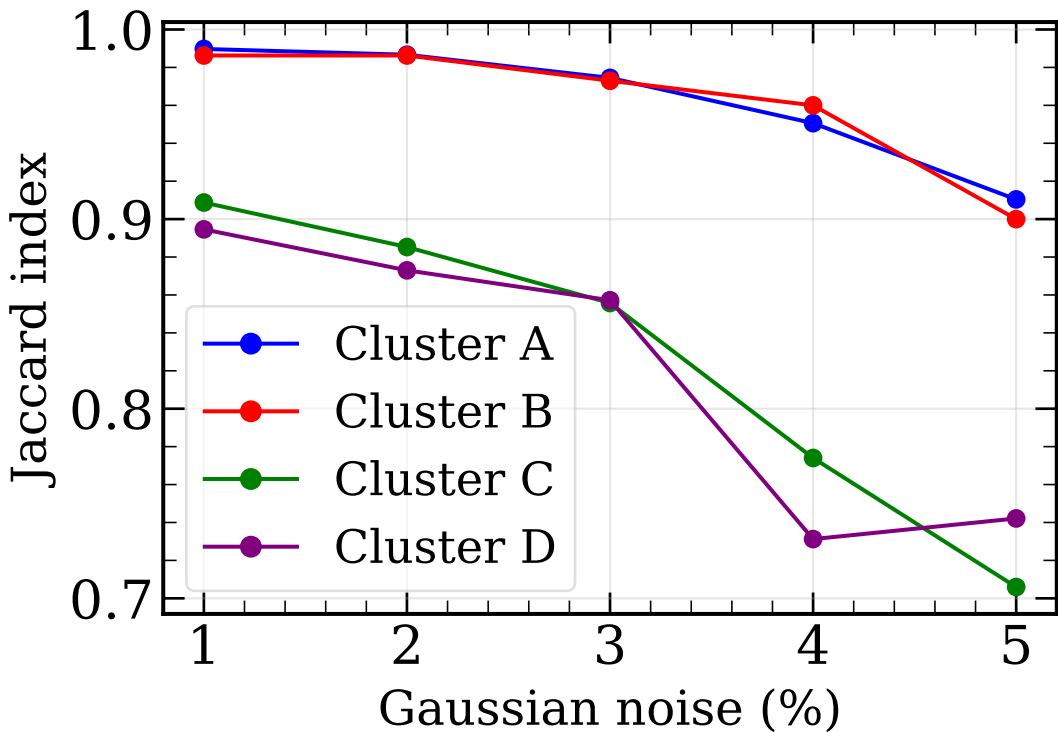

Supplement: S1 Fig — The consistently high JI values, particularly for clusters A and B, demonstrate the robustness of the DSC clustering method against noise perturbations. (PDF) [file pcbi.1013277.s002.pdf]

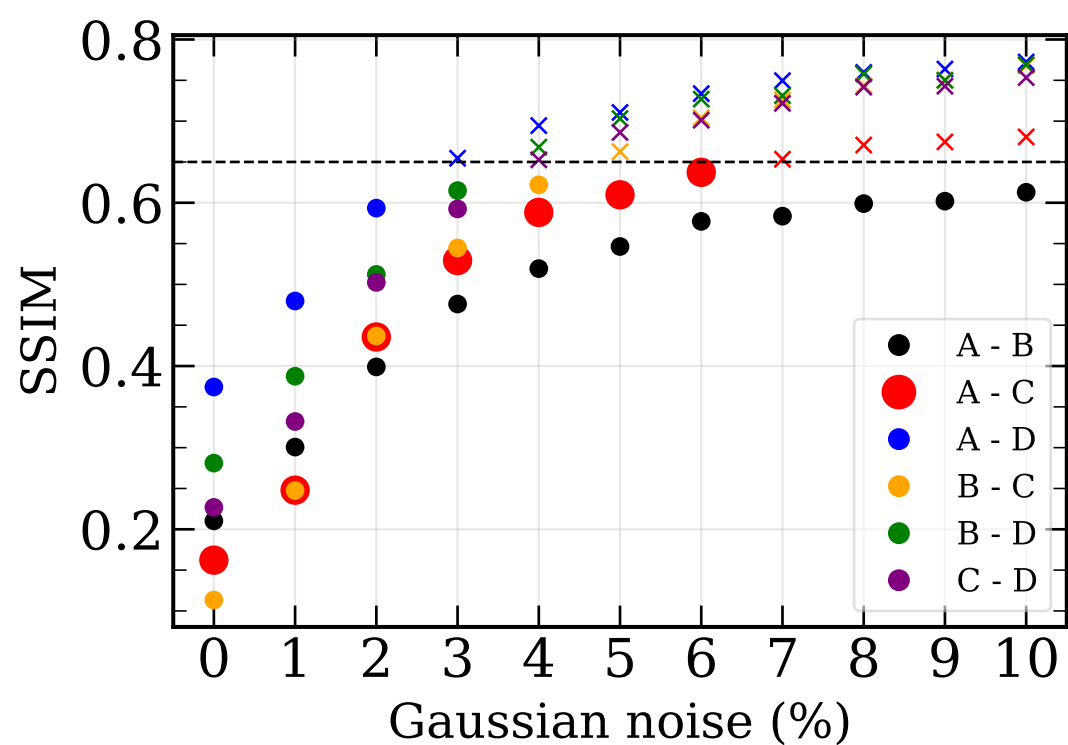

Supplement: S2 Fig — Clusters above the 0.65 SSIM threshold (horizontal dashed line) were indistinguishable to HDSC (× marker). All clusters remained distinct below 3 % noise; at 3 %, clusters A–D merged, and at 4 %, clusters C–D merged, leaving two clusters (A, B) stable up to ∼10 % noise. (PDF) [file pcbi.1013277.s003.pdf]

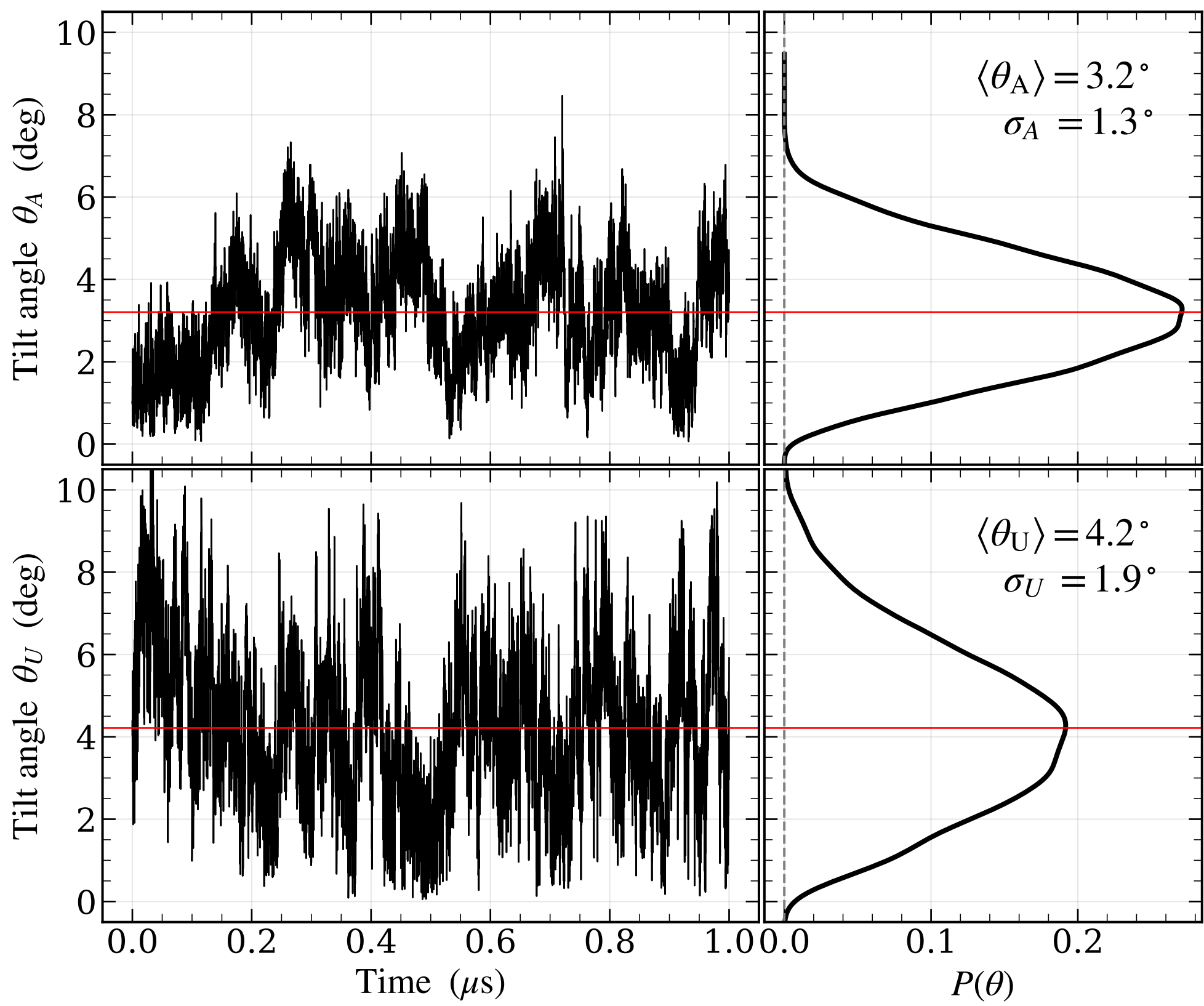

Supplement: S3 Fig — Top panels: aligned [A] trajectory; bottom panels: unaligned [U] trajectory. Left: time series θ(t); right: distributions P(θ). The mean and standard deviation of θ are slightly larger for [U] than for [A], with both distributions being nearly normal. (PDF) [file pcbi.1013277.s004.pdf]
